# The Application of Artificial Intelligence and Machine Learning in Pituitary Adenomas

**DOI:** 10.3389/fonc.2021.784819

**Published:** 2021-12-23

**Authors:** Congxin Dai, Bowen Sun, Renzhi Wang, Jun Kang

**Affiliations:** ^1^ Department of Neurosurgery, Beijing Tongren Hospital, Capital Medical University, Beijing, China; ^2^ Department of Neurosurgery, Peking Union Medical College Hospital, Chinese Academy of Medical Sciences and Peking Union Medical College, Beijing, China

**Keywords:** pituitary adenomas, artificial intelligence, machine learning, radiomics, individualized treatment

## Abstract

Pituitary adenomas (PAs) are a group of tumors with complex and heterogeneous clinical manifestations. Early accurate diagnosis, individualized management, and precise prediction of the treatment response and prognosis of patients with PA are urgently needed. Artificial intelligence (AI) and machine learning (ML) have garnered increasing attention to quantitatively analyze complex medical data to improve individualized care for patients with PAs. Therefore, we critically examined the current use of AI and ML in the management of patients with PAs, and we propose improvements for future uses of AI and ML in patients with PAs. AI and ML can automatically extract many quantitative features based on massive medical data; moreover, related diagnosis and prediction models can be developed through quantitative analysis. Previous studies have suggested that AI and ML have wide applications in early accurate diagnosis; individualized treatment; predicting the response to treatments, including surgery, medications, and radiotherapy; and predicting the outcomes of patients with PAs. In addition, facial imaging-based AI and ML, pathological picture-based AI and ML, and surgical microscopic video-based AI and ML have also been reported to be useful in assisting the management of patients with PAs. In conclusion, the current use of AI and ML models has the potential to assist doctors and patients in making crucial surgical decisions by providing an accurate diagnosis, response to treatment, and prognosis of PAs. These AI and ML models can improve the quality and safety of medical services for patients with PAs and reduce the complication rates of neurosurgery. Further work is needed to obtain more reliable algorithms with high accuracy, sensitivity, and specificity for the management of PA patients.

## Introduction

Pituitary adenomas (PAs) account for approximately 10%–15% of all intracranial neoplasms and are the second most common primary brain tumors (1). PAs are a group of tumors with complex and heterogeneous clinical manifestations that can be classified based on hormone secretion status, clinical features, and radiologic and pathological results. Some PAs are microadenomas (<10 mm) but secrete excess hormones, whereas others are invasive giant PAs (≥40 mm), leading to mass effects but without excessive hormone secretion. Some PAs are asymptomatic and remain stable with long-term follow-up, but others have obvious clinical symptoms at initial diagnosis and need to be treated in a timely manner (2). A subset of PAs is responsive to surgery, medical therapy, and radiotherapy, while others do not respond to these treatments. After standard treatment, some benign PAs achieve long-term remission, whereas other aggressive PAs are refractory to conventional treatments and recur (3). Therefore, it is essential to accurately diagnose PAs early, individually manage PAs, precisely predict the response to treatments, and predict the outcomes of patients with PAs. However, there currently exist no clinical models that can accurately predict the early diagnosis, therapeutic response, and outcomes of patients with PAs.

## Artificial Intelligence and Machine Learning

Artificial intelligence (AI) is a methodology of computer systems that uses algorithms to tirelessly process data, automatically learn and understand its meaning, generate computer models, and identify the best predictive features present in training data (4). As a domain of AI, machine learning (ML) is defined as performing automated learning from the input or data (experience) that it has been presented, and it converts these data to expertise or knowledge. ML can be used to design and train software algorithms to learn from and act on data (5). ML has gained wide applicability to develop sophisticated tools in various areas of data processing, such as images, natural language processing, data mining, gaming, robotics, and big data in general (6). In the past few years, the applications of AI and ML in the healthcare sector have shown ever-increasing growth owing to the rapid progress made possible by deep ML (7).

## Applications of AI and ML in PAs

With the rapid advancement of computer technology, AI and ML have more widely been used in the diagnosis and management of patients with PAs. AI and ML have seen a resurgence with specific application areas in PAs, which involve radiomics, facial imaging, pathological images, electronic medical records including texts, and medical image analyses (6).

## Magnetic Resonance Imaging-Based Radiomics and ML in PAs

As one of the standard examination methods, magnetic resonance imaging (MRI) has been considered one of the most useful tools for detecting PAs. MRI-based radiomics and ML have been used for early screening, differential diagnosis, grading and staging of tumors, clinical decision-making, predicting outcomes, and identifying pathological subtypes (8, 9). The details of the reviewed studies are summarized in **Table 1**. Acromegaly is usually caused by a pituitary growth hormone (GH)-secreting adenoma. Transsphenoidal surgery (TSS) is the first-choice treatment for acromegaly, and tumor consistency is one of the important factors that affect the surgical resection rate. Therefore, it is pivotal to predict the tumor consistency before surgery and to identify individualized surgical strategies for patients with acromegaly. Fan and colleagues (10) enrolled 158 patients with acromegaly and randomly divided them into primary cohort (*n* = 100) and validation cohort (*n* = 58). The preoperative clinical characteristics were collected, and the consistency of the tumor was classified as soft or firm according to the neurosurgeon’s evaluation. The most valuable clinical characteristics were then selected based on the multivariable logistic regression analysis. The critical radiomics features were determined using the elastic net feature selection algorithm, and the radiomics signature was established based on the radiomics features selected from the primary cohort through the support vector machine method. Furthermore, differences in the signature distribution between soft and firm tumors were compared using a violin plot. The radiomics model was then obtained to precisely predict tumor consistency, and the AUC was 0.83 (95% confidence interval, 0.81–0.85) and 0.81 (95% confidence interval, 0.78–0.83) in the primary and validation cohorts, respectively. The authors found that radiomics model is more effective in the prediction of the tumor consistency comparing with the clinical characteristics. Zeynalova and colleagues (11) also enrolled 55 patients with 13 hard and 42 soft pituitary macroadenomas and used an open-source Python package named PyRadiomics for texture feature extraction from coronal T2-weighted original. They reduced the high dimensionality of the histogram texture features with reproducibility analysis, collinearity analysis, and feature selection. Reference standard (hard versus soft) for the classifications of macroadenomas was based on surgical and histopathological findings. The artificial neural network using multilayer perceptron algorithm was utilized for classifications. The authors found that using the ML-based histogram analysis, about three-fourths of pituitary macroadenomas can be correctly classified in term of tumor consistency with an AUC value of 0.710. Furthermore, the ML-based histogram analysis performed better than the signal intensity ratio (SIR) evaluation with an AUC value of 0.551. They indicated that ML-based T2-weighted MRI histogram analysis might be a better technique in predicting the consistency of pituitary macroadenomas than that of conventional SIR evaluation.

**Table 1:** Summary of recent studies related to artificial intelligence and machine learning applications in the pituitary adenomas.

Author and ref.	Tumor subtypes	Sample size	Task	Models (parameters)	Prediction performance (AUC)	
Fan et al. (10)	Acromegaly	Training (*n* = 100) Test datasets (*n* = 58)	Predicting consistency	Elastic net feature selection algorithm	0.83	
Zeynalova et al. (11)	Pituitary macroadenoma	*N* = 55	Predicting consistency	Artificial neural network	0.710	
Zhu et al. (12)	PAs	*N* = 152	Determining the softness	CRNN (DenseNet+ResNet)	0.9178	
Niu et al. (13)	PAs	Training set (*n* = 97) Test set (*n* = 97)	Predicting CSI	Linear support vector machine and nomogram	Training (0.899) Test (0.871)	
Fan et al. (14)	Invasive functional PAs	Primary (*n* = 108) Validation (*n* = 55)	Predicting treatment response	Support vector machine	Training (0.832) Validation (0.811)	
Staartjes et al. (15)	PAs	*N* = 140	Predicting gross-total resection	Deep neural network	0.96	
Fan et al. (16)	Acromegaly	Training (*n* = 534) Test datasets (*n* = 134)	Predicting TSS response	Forward search algorithm	Training (0.8555) Validation (0.8178)	
Qiao et al. (17)	Acromegaly	Training (*n* = 833)	Test datasets (*n* = 99) Predicting early remission of TSS	Partial model, full model	Partial model	Full model
				Penalized logistic regression	0.781	0.867
				Gradient boost machine	0.752	0.789
				Support vector machine	0.759	0.850
				Neural network	0.790	0.787
				Ensemble algorithm	0.775	0.853
					Validation	cohort
					0.759	0.897
Hollon et al. (18)	PAs	Training (*n* = 300) Test datasets (*n* = 100)	Predicting early outcomes	Naive Bayes	0.795	
				Support vector machines	0.826	
				Random forest	0.848	
				LR-EN regularization	0.827	
Dai et al. (19)	Acromegaly	Training (*n* = 244) Test dataset (*n* = 62)	Predicting delayed remission	Logistic regression	0.7945	
				Adaptive boosting	0.7013	
				GBDT	0.8061	
				Extreme gradient boost	0.8260	
				Categorical boosting	0.8239	
				Random forest	0.7338	
Fan et al. (20)	Acromegaly	*N* = 57	Predicting radiotherapeutic response	Support vector machine	0.96	
Kocak et al. (21)	Acromegaly	*N* = 47	Predicting response to SA	Wrapper-based algorithm	0.847	
Park et al. (22)	Prolactinoma	Training (*n* = 141) Test dataset (*n* = 36)	Predicting the DA response	Random forest	0.78 (0.63–0.94)	
				Extra-trees	0.79 (0.63–0.95)	
				Light GBM	0.74 (0.57–0.93)	
				QDA	0.66 (0.48–0.84)	
				LDA	0.66 (0.46–0.86)	
				Soft voting ensemble	0.81 (0.67–0.96)	
Zoli et al. (23)	Cushing disease	Training (*n* = 121) Test dataset (*n* = 30)	Predicting outcomes of TSS		Training and test	
				Support vector machine	0.681 and 1.00	
				GBM	0.719 and 0.783	
				K-nearest neighbor	0.993 and 0.988	
Zhang et al. (24)	Cushing disease	Training (*n* = 836) Test dataset (*n* = 209)	Predicting postoperative immediate remission	Extreme gradient boost	0.712	
				GBDT	0.734	
				Random forest	0.726	
				Adaptive boost	0.699	
				Naïve Bayes	0.681	
				Logistic regression	0.701	
				Decision tree	0.664	
				Multilayer perceptron	0.700	
				Stacking	0.743	
Fan et al. (25)	Cushing disease	Training (*n* = 836) Test dataset (*n* = 209)	Predicting Postoperative Delayed remission	Logistic regression	0.7262	
				Adaptive boosting	0.7619	
				GBDT	0.7262	
				XGboost	0.7262	
				Catboost	0.7	
Liu et al. (26)	Cushing disease	Training (*n* = 283) Test dataset (*n* = 71)	Predicting recurrence after TSS	Decision tree	0.629	
				Random forest	0.779	
				Logistic regression	0.684	
				Naïve Bayes	0.608	
				GBDT	0.694	
				Adaptive boost	0.716	
				Extreme gradient boost	0.735	
Voglis et al. (27)	PAs	*N* = 207	Predicting postoperative hyponatremia	Random forest	0.637	
				Naïve Bayes	0.646	
				Boosted GLMs	0.671	
				GLMs	0.595	
Machado et al. (28)	NFP macroadenomas	*N* = 27	Predicting recurrence after the first surgery		2D radiomics	3D radiomics
				Multilayer perceptron	0.92.9	0.962
				Random forest	0.877	0.962
				Support vector machine	0.860	0.946
				Logistic regression (LR)	0.929	0.946
				K-nearest neighbor	0.979	0.945
Meng et al. (29)	Acromegaly	62 patients with acromegaly and 62 matched controls	Identifying facial features and predicting patients of acromegaly	Linear discriminant analysis	0.9286	
Wei et al. (30)	Acromegaly and Cushing disease	642 Cushing disease, 896 acromegaly, and 11,447 normal images	Identifying facial anomalies	Convolutional neural networks	Cushing disease	0.9647
					Acromegaly	0.9556
					Normal	0.9393
Peng et al. (31)	PAs	235 patients with pathologically diagnosed PAs	Immunohistochemically classify PAs subtypes	Support vector machine	0.9549	
				K-nearest neighbor	0.9266	
				Naïve Bayes	0.932	
Ugga et al. (32)	PAs	89 patients with available Ki-67 labeling index	Predicting of high proliferative index	K-nearest neighbors	0.87	

Zhu and colleagues (12) also used 152 patient data with labels (including 112 T1 MRI spatial sequences and 40 T2 MRI spatial sequences) and presented an automatic method for accurately determining the softness level of pituitary tumors preoperatively. Because their pituitary tumor MRI image dataset where T1 and T2 sequence data are unbalanced (due to data missing) and undersampled. They first obtained fully sampled MRI spatial sequence by using a CycleConsistent Adversarial Networks (CycleGAN) model. They then used a Densely Connected Convolutional Networks (DenseNet)-Deep Residual Networks (ResNet)-based Autoencoder framework to optimize the feature extraction process for pituitary tumor image data. Finally, they used a Convolutional Recurrent Neural Network (CRNN) model to classify pituitary tumors based on their predicted softness levels. They found that this semisupervised deep neural network model can accurately determine the softness level of pituitary tumors with high accuracy (91.78%).

Although these ML-based radiomics have been shown a very high accuracy in predicting consistency of the pituitary tumors, each approach has its own pros and cons. Firstly, all the studies were a retrospective analysis with a relatively small number of patients, which would lead to bias in ML-based classifications. Secondly, Zeynalova and colleagues (11) only used histogram analysis with few texture features, two dimensional segmentations, and conventional T2-weighted MRI, which were not comprehensive. More ML and feature selection algorithms and more comprehensive MRI data including contrast-enhanced MRI scans may have a potential for developing better ML-based models. Thirdly, the data samples used in Zhu’s (12) study were unbalanced sequence image data and insufficient; it is easy to produce the overfitting phenomenon. Although the loss of feature extraction model training was low and convergence was achieved, the accuracy was still not high enough. Taken together, these ML-based radiomics models performed better than conventional methods in predicting the consistency of the pituitary tumors; further large-scale and more comprehensive studies are needed to confirm and improve these approaches.

Invasive PAs are complicated and difficult to treat; therefore, it is critical to predict cavernous sinus (CS) invasion and treatment response for these patients preoperatively. Niu and colleagues (13) predicted CS invasion preoperatively for patients with Knosp grades II and III PAs using a radiomics method based on MR, which might contribute to designing surgical strategies. Fan and colleagues (14) developed and validated a radiomic model incorporating an MRI-based radiomic signature using a support vector machine, which predicts the treatment response and help doctors determine individual treatment strategies for these patients with invasive functional PAs. Gross-total resection is often the primary surgical goal in TSS for PAs. Staartjes and colleagues (15) demonstrated that a deep ML model could be used to preoperatively predict the likelihood of GTR with excellent performance, which would be a valuable addition to risk stratification and surgical decision-making. Accurate prediction of postoperative remission may be helpful for decision-making and prognosis regarding treatment strategies for patients with acromegaly. Fan and colleagues (16) enrolled 668 patients with acromegaly and divided them into a training set of 534 cases and a test set of 134 cases. The author used six machine learning methods in Python, including random forest, logistic regression, logistic GAMs, gradient boosting decision tree (GBDT), adaptive boosting, and extreme gradient boost, to construct a predictive model for postoperative remission. By comparing the six models, the GBDT model has the best predictive performance, can obtain quantitative predictive value, has a higher accuracy rate than clinicians, and can better assist the preoperative clinical diagnosis and treatment decision-making of patients with acromegaly. Qiao (17) included 833 patients with GH-secreting PAs as a training cohort and trained a partial model (using only preoperative variables) and a full model (using all variables) to predict off-medication endocrine remission at the 6-month follow-up after TSS using multiple ML algorithms. These models have been validated to accurately predict early endocrine remission after TSS in patients with GH-secreting PAs. The prediction accuracy of the ML-trained models was better than those using single variables. Hollon (18) also demonstrated that early surgical outcomes of PAs can be predicted with 87% accuracy using a machine learning approach.

For patients with acromegaly who do not reach immediate remission after surgery, a subset of them achieves delayed remission during long-term follow-up without further postoperative therapy. Therefore, it is necessary to predict the delayed remission of acromegaly after surgery (33). We used the recursive feature elimination algorithm to select features and applied six ML algorithms to establish an ML model for predicting delayed remission of acromegaly. As an effective noninvasive approach, ML-based models can predict delayed remission and aid in determining individual treatment and follow-up strategies for patients with acromegaly who have not achieved remission within 6 months of surgery (19).

For acromegaly patients who do not achieve remission after TSS, radiotherapy is a third-line treatment. Fan and colleagues developed a radiomics model using preradiotherapy clinical and MRI data to noninvasively predict the radiotherapeutic response of acromegaly, which may help doctors identify acromegaly patients who will benefit from radiotherapy (20). Somatostatin analogs (SAs) are widely used in the medical treatment of patients with acromegaly, and it is necessary to predict the response to SA for these patients. Kocak (21) demonstrated that ML-based high-dimensional quantitative texture analysis on T2-weighted MRI has the potential to predict the response to SAs in patients with acromegaly, and it performs better than quantitative and qualitative T2-weighted relative signal intensity or immunohistochemical granulation pattern evaluation.

For prolactinomas, medical treatment with dopamine agonists (DAs) is the first-line therapy. However, approximately 10%–30% of patients with prolactinomas show resistance to DA (34). Therefore, it is crucial to identify DA-resistant prolactinomas early because then the patients would not have to endure a prolonged therapeutic trial. Park (22) developed a radiomics model using an ensemble machine learning classifier with conventional MRIs and demonstrated that radiomics features might be useful biomarkers to predict the DA response in patients with prolactinoma.

Cushing disease (CD) is a devastating condition that is usually caused by excessive secretions arising from pituitary corticotroph adenomas (35). It remains challenging to accurately diagnose and individually manage CD due to the disease complexity and heterogeneity (36). It is especially important to preoperatively predict the treatment outcomes of these patients due to variable rates of remission and a high risk of recurrence (37). In recent years, AI and ML have been increasingly reported in the diagnosis and management of CD (38). TSS is the first-line treatment for patients with CD; however, surgical outcomes are usually the most difficult to predict preoperatively. Zoli and colleagues (23) trained and internally validated robust models using ML algorithms to make accurate preoperative surgical outcome predictions for CD patients. Zhang (24) also developed a readily available ML-based model for the preoperative prediction of immediate remission in patients with histology-positive CD. After TSS, a subset of patients with CD do not achieve immediate remission but achieve remission without further postoperative therapy during long-term follow-up, which is defined as postoperative delayed remission (39). Among these patients with persistent hypercortisolism after TSS, some patients will achieve delayed remission without the need for further treatment. To identify patients who have the potential to achieve delayed remission, Fan (25) developed ML-based models to predict delayed remission or persistent active disease in patients with CD whose remission status is uncertain. Use of this model could help doctors judge the surgical response and determine whether the patient needs postoperative adjuvant therapy, thus avoiding unnecessary additional treatments. According to previous studies, recurrence after TSS for CD ranges from 15% to 66% (40), whereas no valid predictor for recurrence has been developed. Liu (26) reported that using ML-based models was feasible for predicting CD recurrence after initial TSS, which was significantly better than that of some conventional models.

After TSS, postoperative hyponatremia is one of the common procedural complications in patients with PAs. Voglis (27) demonstrated that a trained ML model was able to learn complex risk factor interactions and could predict postoperative hyponatremia, thus potentially reducing morbidity and improving patient safety.

After the first surgery, 12% to 66% of patients with clinically nonfunctioning pituitary adenoma (NFPA) experience a tumor recurrence. Nevertheless, there is still no factor that could concisely predict the recurrence of NFPA. Machado (28) reported that a combination of radiomics with machine-learning algorithms could offer computational models capable of noninvasive, unbiased, and quick assessment that might improve the prediction of NFPA recurrence.

Taken together, these ML-based and MRI-based radiomics analytical methods are playing an increasingly important role in early accurate diagnosis, individualized treatment, predicting the response to treatments, including surgery, medications and radiotherapy, and the prognosis of patients with PAs. However, there is significant variability in the applied ML paradigms and prediction performance (AUC) at different studies (**Table 1**). The main reasons for that include variation in data extraction and lack of consistency among the statistical methodologies and ML algorithms used in the varied studies. In the future, ML-based and MRI-based radiomics will have great promise for potentially improving patients’ individualized treatment and prognosis.

## Facial Imaging-Based AI and ML in PAs

Facial changes are common among nearly all patients with acromegaly and CD. It is difficult to notice such facial changes early because they are a slow and gradual process. The diagnosis and treatment of these diseases are often delayed until these clinical symptoms become obvious. Meng (29) demonstrated that combining 3D imaging and ML techniques could accurately identify and predict early facial changes in patients with acromegaly, which might be beneficial for the early detection of acromegalic patients, enabling immediate treatment. Wei (30) also developed a deep-learning model to recognize facial anomalies with underlying endocrine disorders, and its performance was comparable with that of professional medical practitioners. These models have the potential to assist in the diagnosis and follow-up of these patients with hypersecretion statuses, which may be helpful for the early detection of the disease.

## Pathological Pictures-Based AI and ML in PAs

The type of PA cannot be clearly recognized by preoperative MRI but can be classified by immunohistochemical staining of resected tumor samples after surgery. Recently, PAs have been classified based on a combination of tumor hormonal content and pituitary transcription factors. The correct PA classification before surgery can help doctors decide on the right treatment strategy. Peng (31) developed a classification model using ML-based radiomics, which can potentially precisely immunohistochemically classify PA subtypes. This model exhibited good performance and might offer potential guidance to doctors in clinical decision-making before surgery. The Ki-67 labeling index, representing a proliferative marker, has been reported as a marker of aggressiveness in PAs (41), and it is crucial to identify the Ki-67 labeling index early to allow timely diagnosis and treatment. Ugga and colleagues (32) proved that ML analysis of texture-derived parameters from preoperative T2 MRI could effectively predict the Ki-67 proliferation index class in pituitary macroadenomas. This might provide a more accurate preoperative lesion classification for doctors before surgery and help neurosurgeons develop surgical strategies.

## Surgical Microscopic Video-Based AI and ML in PAs

In pituitary surgery, segmentation of the surgical workflow might be helpful for providing context-sensitive user interfaces or generating automatic reports. Moreover, neurosurgeons must deal with intraoperative adverse events, which come from not only the patients but also surgical management. It is very important to be aware of these difficulties quickly and efficiently, to better handle risky situations and to relieve the neurosurgeons’ responsibilities. It is necessary to assist neurosurgeries through the understanding of operating room activities, increase medical safety, and support decision-making. Lalys (42) recognized surgical phases of every unknown image by computing their signatures and then simulating them with machine learning techniques and validated this methodology with a specific type of neurosurgery. Currently, this methodology could be used for postoperative video indexation as an aid to surgeons, which contains relevant surgical phases of each procedure for easy browsing.

## Total Charges and Drivers of Cost in PAs

The effective allocation of resources in the healthcare system enables providers to care for an increasing number of needier patients. It is necessary to identify drivers of total charges for TSS for PAs, which may help neurosurgeons reduce waste and provide higher-quality care for patients. Muhlestein and colleagues (43) used a large, national database to develop ML ensembles that directly predict total charges for PA patients with good fidelity. They identified extended length of stay, postoperative complications, private investor hospital ownership, etc. as drivers of total charges and potential targets for cost-lowering interventions. Minimizing the effects of these variables may improve efficiency in the resource-limited healthcare system and lead to higher-quality care and improved outcomes for more patients.

## Future Perspectives of AI and ML in PAs

To date, AI and ML are promising in the diagnosis, prediction of therapy response, and prognosis, as well as the pathological classification of PAs. AI-based radiomics has especially made the greatest contributions to bridging the gap of AI-assisted diagnostics and prognostics to individualized treatment. However, the sample sizes included in the previous studies were relatively small, and the accuracy of the algorithms is not yet very high. Therefore, future studies including larger sample sizes may obtain more reliable algorithms with high accuracy, sensitivity, and specificity. Currently, there is a lack of consistency among the statistical methodologies and ML algorithms incorporated by the studies described. The wide variety of methodologies and ML models always leads to inconsistent conclusions. Given this lack of standardization, a consensus is required to standardize the extrapolation of data and model development. Moreover, it appears that there are many aspects for future researchers to include contributions of AI and ML in PAs. First, it is important to accurately predict the disability and mortality risks among patients with PAs using ML algorithms. Accurately predicting these risks, such as heart failure risk in patients with acromegaly and fracture risk in patients with CD, enables an individualized approach to prevention, monitoring, and therapy strategies. Second, there is currently a lack of healthcare policy generated by AI technologies on PAs. Making appropriate medical policies by analyzing big data from public healthcare using AI technologies would be helpful to improve the accuracy and personalized medical care of the entire medical community. Third, more interdisciplinary studies are necessary to strengthen AI links with medical big data management and enable the creation of publicly available datasets for neuroimaging- and visual imaging-guided diagnosis and treatment of PAs.

## Conclusions

As an emerging field, AI and ML method research has displayed great prospects in patients with PAs. The current use of AI and ML models has the potential to assist doctors and patients in making crucial surgical decisions by providing an accurate diagnosis and predicting the response to treatment and the outcomes of PAs. These AI and ML models have more individual specificity and accuracy than traditionally used models, and AI-based clinical decision support systems are likely to improve further the quality and safety of medical services for patients with PAs and reduce the complication rates of neurosurgery. Additional work is necessary to obtain more reliable algorithms with high accuracy, sensitivity, and specificity for the management of PA patients.

## Author Contributions

CD and BS revised the manuscript. RW and JK take final responsibility for this article. All authors listed have made substantial, direct, and intellectual contribution to the work and approved it for publication.

## Funding

Financial support for this study was provided by the Beijing Municipal Administration of Hospitals Incubating Program, (grant number: PX2022004). The funding institutions had no role in the design of the study, data collection and analysis, decision to publish, or preparation of the manuscript.

## Conflict of Interest

The authors declare that the research was conducted in the absence of any commercial or financial relationships that could be construed as a potential conflict of interest.

## Publisher’s Note

All claims expressed in this article are solely those of the authors and do not necessarily represent those of their affiliated organizations, or those of the publisher, the editors and the reviewers. Any product that may be evaluated in this article, or claim that may be made by its manufacturer, is not guaranteed or endorsed by the publisher.

## References

[1] Araujo-Castro M Berrocal VR Pascual-Corrales E. Pituitary Tumors: Epidemiology and Clinical Presentation Spectrum. Hormones (Athens) (2020) 19(2):145–55. doi: 10.1007/s42000-019-00168-8 31933100

[2] Dai C Kang J Liu X Yao Y Wang H Wang R. How to Classify and Define Pituitary Tumors: Recent Advances and Current Controversies. Front Endocrinol (Lausanne) (2021) 12:604644. doi: 10.3389/fendo.2021.604644 33815274 PMC8010908

[3] Dai C Liu X Ma W Wang R. The Treatment of Refractory Pituitary Adenomas. Front Endocrinol (2019) 10:334. doi: 10.3389/fendo.2019.00334 PMC6548863 31191457

[4] Gurgitano M Angileri SA Roda GM Liguori A Pandolfi M Ierardi AM. Interventional Radiology Ex-Machina: Impact of Artificial Intelligence on Practice. Radiol Med (2021) 126(7):998–1006. doi: 10.1007/s11547-021-01351-x 33861421 PMC8050998

[5] Hong N Park H Rhee Y. Machine Learning Applications in Endocrinology and Metabolism Research: An Overview. Endocrinol Metab (Seoul) (2020) 35(1):71–84. doi: 10.3803/EnM.2020.35.1.71 32207266 PMC7090299

[6] Saha A Tso S Rabski J Sadeghian A Cusimano MD. Machine Learning Applications in Imaging Analysis for Patients With Pituitary Tumors: A Review of the Current Literature and Future Directions. Pituitary (2020) 23(3):273–93. doi: 10.1007/s11102-019-01026-x 31907710

[7] Secinaro S Calandra D Secinaro A Muthurangu V Biancone P. The Role of Artificial Intelligence in Healthcare: A Structured Literature Review. BMC Med Inform Decis Mak (2021) 21(1):125. doi: 10.1186/s12911-021-01488-9 33836752 PMC8035061

[8] Fan Y Feng M Wang R. Application of Radiomics in Central Nervous System Diseases: A Systematic Literature Review. Clin Neurol Neurosurg (2019) 187:105565. doi: 10.1016/j.clineuro.2019.105565 31670024

[9] Soldozy S Farzad F Young S Yagmurlu K Norat P Sokolowski J. Pituitary Tumors in the Computational Era, Exploring Novel Approaches to Diagnosis, and Outcome Prediction With Machine Learning. World Neurosurg (2021) 146:315–21.e1. doi: 10.1016/j.wneu.2020.07.104 32711142

[10] Fan Y Hua M Mou A Wu M Liu X Bao X. Preoperative Noninvasive Radiomics Approach Predicts Tumor Consistency in Patients With Acromegaly: Development and Multicenter Prospective Validation. Front Endocrinol (Lausanne) (2019) 10:403. doi: 10.3389/fendo.2019.00403 31316464 PMC6611436

[11] Zeynalova A Kocak B Durmaz ES Comunoglu N Ozcan K Ozcan G. Preoperative Evaluation of Tumour Consistency in Pituitary Macroadenomas: A Machine Learning-Based Histogram Analysis on Conventional T2-Weighted MRI. Neuroradiology (2019) 61(7):767–74. doi: 10.1007/s00234-019-02211-2 31011772

[12] Zhu H Fang Q Huang Y Xu K. Semi-Supervised Method for Image Texture Classification of Pituitary Tumors *via* CycleGAN and Optimized Feature Extraction. BMC Med Inform Decis Mak (2020) 20(1):215. doi: 10.1186/s12911-020-01230-x 32907561 PMC7488038

[13] Niu J Zhang S Ma S Diao J Zhou W Tian J. Preoperative Prediction of Cavernous Sinus Invasion by Pituitary Adenomas Using a Radiomics Method Based on Magnetic Resonance Images. Eur Radiol (2019) 29(3):1625–34. doi: 10.1007/s00330-018-5725-3 PMC6510860 30255254

[14] Fan Y Liu Z Hou B Li L Liu X Liu Z. Development and Validation of an MRI-Based Radiomic Signature for the Preoperative Prediction of Treatment Response in Patients With Invasive Functional Pituitary Adenoma. Eur J Radiol (2019) 121:108647. doi: 10.1016/j.ejrad.2019.108647 31561943

[15] Staartjes VE Serra C Muscas G Maldaner N Akeret K van Niftrik C. Utility of Deep Neural Networks in Predicting Gross-Total Resection After Transsphenoidal Surgery for Pituitary Adenoma: A Pilot Study. Neurosurg Focus (2018) 45(5):E12. doi: 10.3171/2018.8.FOCUS18243 30453454

[16] Fan Y Li Y Li Y Feng S Bao X Feng M. Development and Assessment of Machine Learning Algorithms for Predicting Remission After Transsphenoidal Surgery Among Patients With Acromegaly. Endocrine (2020) 67(2):412–22. doi: 10.1007/s12020-019-02121-6 31673954

[17] Qiao N Shen M He W He M Zhang Z Ye H. Machine Learning in Predicting Early Remission in Patients After Surgical Treatment of Acromegaly: A Multicenter Study. Pituitary (2021) 24(1):53–61. doi: 10.1007/s11102-020-01086-4 33025547

[18] Hollon TC Parikh A Pandian B Tarpeh J Orringer DA Barkan AL. A Machine Learning Approach to Predict Early Outcomes After Pituitary Adenoma Surgery. Neurosurg Focus (2018) 45(5):E8. doi: 10.3171/2018.8.FOCUS18268 30453460

[19] Dai C Fan Y Li Y Bao X Li Y Su M. Development and Interpretation of Multiple Machine Learning Models for Predicting Postoperative Delayed Remission of Acromegaly Patients During Long-Term Follow-Up. Front Endocrinol (Lausanne) (2020) 11:643. doi: 10.3389/fendo.2020.00643 33042013 PMC7525125

[20] Fan Y Jiang S Hua M Feng S Feng M Wang R. Machine Learning-Based Radiomics Predicts Radiotherapeutic Response in Patients With Acromegaly. Front Endocrinol (Lausanne) (2019) 10:588. doi: 10.3389/fendo.2019.00588 31507537 PMC6718446

[21] Kocak B Durmaz ES Kadioglu P Polat KO Comunoglu N Tanriover N. Predicting Response to Somatostatin Analogues in Acromegaly: Machine Learning-Based High-Dimensional Quantitative Texture Analysis on T2-Weighted MRI. Eur Radiol (2019) 29(6):2731–9. doi: 10.1007/s00330-018-5876-2 30506213

[22] Park YW Eom J Kim S Kim H Ahn SS Ku CR. Radiomics With Ensemble Machine Learning Predicts Dopamine Agonist Response in Patients With Prolactinoma. J Clin Endocrinol Metab (2021) 106(8):e3069–77. doi: 10.1210/clinem/dgab159 33713414

[23] Zoli M Staartjes VE Guaraldi F Friso F Rustici A Asioli S. Machine Learning-Based Prediction of Outcomes of the Endoscopic Endonasal Approach in Cushing Disease: Is the Future Coming? Neurosurg Focus (2020) 48(6):E5. doi: 10.3171/2020.3.FOCUS2060 32480364

[24] Zhang W Sun M Fan Y Wang H Feng M Zhou S. Machine Learning in Preoperative Prediction of Postoperative Immediate Remission of Histology-Positive Cushing’s Disease. Front Endocrinol (Lausanne) (2021) 12:635795. doi: 10.3389/fendo.2021.635795 33737912 PMC7961560

[25] Fan Y Li Y Bao X Zhu H Lu L Yao Y. Development of Machine Learning Models for Predicting Postoperative Delayed Remission in Patients With Cushing’s Disease. J Clin Endocrinol Metab (2021) 106(1):e217–31. doi: 10.1210/clinem/dgaa698 33000120

[26] Liu Y Liu X Hong X Liu P Bao X Yao Y. Prediction of Recurrence After Transsphenoidal Surgery for Cushing’s Disease: The Use of Machine Learning Algorithms. Neuroendocrinology (2019) 108(3):201–10. doi: 10.1159/000496753 30630181

[27] Voglis S van Niftrik C Staartjes VE Brandi G Tschopp O Regli L. Feasibility of Machine Learning Based Predictive Modelling of Postoperative Hyponatremia After Pituitary Surgery. Pituitary (2020) 23(5):543–51. doi: 10.1007/s11102-020-01056-w 32488759

[28] Machado LF Elias P Moreira AC Dos SA Murta JL. MRI Radiomics for the Prediction of Recurrence in Patients With Clinically Non-Functioning Pituitary Macroadenomas. Comput Biol Med (2020) 124:103966. doi: 10.1016/j.compbiomed.2020.103966 32860977

[29] Meng T Guo X Lian W Deng K Gao L Wang Z. Identifying Facial Features and Predicting Patients of Acromegaly Using Three-Dimensional Imaging Techniques and Machine Learning. Front Endocrinol (Lausanne) (2020) 11:492. doi: 10.3389/fendo.2020.00492 32849283 PMC7403213

[30] Wei R Jiang C Gao J Xu P Zhang D Sun Z. Deep-Learning Approach to Automatic Identification of Facial Anomalies in Endocrine Disorders. Neuroendocrinology (2020) 110(5):328–37. doi: 10.1159/000502211 31319415

[31] Peng A Dai H Duan H Chen Y Huang J Zhou L. A Machine Learning Model to Precisely Immunohistochemically Classify Pituitary Adenoma Subtypes With Radiomics Based on Preoperative Magnetic Resonance Imaging. Eur J Radiol (2020) 125:108892. doi: 10.1016/j.ejrad.2020.108892 32087466

[32] Ugga L Cuocolo R Solari D Guadagno E D’Amico A Somma T. Prediction of High Proliferative Index in Pituitary Macroadenomas Using MRI-Based Radiomics and Machine Learning. Neuroradiology (2019) 61(12):1365–73. doi: 10.1007/s00234-019-02266-1 31375883

[33] Wang Z Guo X Gao L Feng C Lian W Deng K. Delayed Remission of Growth Hormone-Secreting Pituitary Adenoma After Transsphenoidal Adenectomy. World Neurosurg (2019) 122:e1137–45. doi: 10.1016/j.wneu.2018.11.004 30447463

[34] Maiter D. Management of Dopamine Agonist-Resistant Prolactinoma. Neuroendocrinology (2019) 109(1):42–50. doi: 10.1159/000495775 30481756

[35] Nishioka H Yamada S. Cushing’s Disease. J Clin Med (2019) 8(11):1951. doi: 10.3390/jcm8111951 PMC6912360 31726770

[36] Serban AL Del SG Sala E Carosi G Indirli R Rodari G. Determinants of Outcome of Transsphenoidal Surgery for Cushing Disease in a Single-Centre Series. J Endocrinol Invest (2020) 43(5):631–9. doi: 10.1007/s40618-019-01151-1 31773581

[37] Braun LT Rubinstein G Zopp S Vogel F Schmid-Tannwald C Escudero MP. Recurrence After Pituitary Surgery in Adult Cushing’s Disease: A Systematic Review on Diagnosis and Treatment. Endocrine (2020) 70(2):218–31. doi: 10.1007/s12020-020-02432-z PMC7396205 32743767

[38] Laws ER Catalino MP. Editorial. Machine Learning and Artificial Intelligence Applied to the Diagnosis and Management of Cushing Disease. Neurosurg Focus (2020) 48(6):E6. doi: 10.3171/2020.3.FOCUS20213 32480365

[39] Hinojosa-Amaya JM Cuevas-Ramos D. The Definition of Remission and Recurrence of Cushing’s Disease. Best Pract Res Clin Endocrinol Metab (2021) 35(1):101485. doi: 10.1016/j.beem.2021.101485 33472761

[40] Nieman LK Biller BM Findling JW Murad MH Newell-Price J Savage MO. Treatment of Cushing’s Syndrome: An Endocrine Society Clinical Practice Guideline. J Clin Endocrinol Metab (2015) 100(8):2807–31. doi: 10.1210/jc.2015-1818 PMC4525003 26222757

[41] Liu X Dai C Feng M Li M Chen G Wang R. Diagnosis and Treatment of Refractory Pituitary Adenomas: A Narrative Review. Gland Surg (2021) 10(4):1499–507. doi: 10.21037/gs-20-873 PMC8102214 33968701

[42] Lalys F Riffaud L Morandi X Jannin P. Automatic Phases Recognition in Pituitary Surgeries by Microscope Images Classification. In: IPCAI 2010: Information Processing in Computer-Assisted Interventions, First International Conference; 2010 June 23. Geneva, Switzerland. Berlin: Springer (2010). p. 34–44. doi: 10.1007/978-3-642-13711-2_4

[43] Muhlestein WE Akagi DS McManus AR Chambless LB. Machine Learning Ensemble Models Predict Total Charges and Drivers of Cost for Transsphenoidal Surgery for Pituitary Tumor. J Neurosurg (2018) 131(2):507–16. doi: 10.3171/2018.4.JNS18306 30239321

